# Immunohistochemical basigin expression level in thyroid cancer tissues

**DOI:** 10.1186/s12957-020-01975-9

**Published:** 2020-09-05

**Authors:** Wan-Ping Guo, Deng Tang, Yu-Yan Pang, Xiao-Jiao Li, Gang Chen, Zhi-Guang Huang, Xiao-Zhun Tang, Qin-Qiao Lai, Jin-Yan Gan, Xiao-Li Huang, Xiao-Fan Liu, Zhi-Xiao Wei, Wei Ma

**Affiliations:** 1 Department of Pathology, First Affiliated Hospital of Guangxi Medical University, 6 Shuangyong Road, Nanning, 530021 Guangxi Zhuang Autonomous Region People’s Republic of China; 2 Department of Nuclear Medicine, First Affiliated Hospital of Guangxi Medical University, 6 Shuangyong Road, Nanning, 530021 Guangxi Zhuang Autonomous Region People’s Republic of China; 3 Department of Head and Neck Tumor Surgery, Guangxi Medical University Cancer Hospital, 71 Hedi Road, Nanning, 530021 Guangxi Zhuang Autonomous Region People’s Republic of China

**Keywords:** Basigin, Thyroid cancer, Thyroid papillary carcinoma, Tissue microarray, RNA-sequencing

## Abstract

**Background:**

Thyroid cancer (TC) is the most common endocrine malignancy; basigin (also known as BSG) plays a crucial role in tumor cell invasion, metastasis, and angiogenesis. This study was designed to identify the change of BSG expression in TC and its possible potential mechanism.

**Methods:**

The BSG expression levels in TC were demonstrated using data collected from in-house immunohistochemical (IHC), RNA-sequencing (RNA-seq), microarrays, and literatures. Integrated analysis was performed to determined BSG expression levels in TC comprehensively. The Gene Ontology (GO) and Kyoto Encyclopedia of Genes and Genomes (KEGG) enrichment analyses were performed with the integration of BSG co-expressed genes and differentially expressed genes (DEGs) in TC tissues to explore the potential mechanisms of BSG in TC.

**Results:**

The protein expression level of BSG was significantly higher in TC cases based on the IHC experiments. In addition, the combined SMD for BSG expression was 0.39 (*p* < 0.0001), the diagnostic odds ratio was 3.69, and the AUC of the sROC curve was 0.6986 using 1182 TC cases and 437 non-cancerous cases from 17 independent datasets. Furthermore, BSG co-expressed genes tended to be enriched in gene terms of the extracellular matrix (ECM), cell adhesion, and cell-cell interactions. The expression levels of nine hub BSG co-expressed genes were markedly upregulated in TC cases.

**Conclusion:**

BSG expression levels were closely correlated with the progression of TC and may affect the signals of the ECM, cell adhesion, and cell-cell interactions.

## Background

Thyroid cancer (TC) is the most common endocrine malignancy, and its prevalence has increased dramatically in the last few years [1–3]. Histological types of TC include papillary carcinoma (PTC), follicular carcinoma (FTC), anaplastic carcinoma (ATC)/undifferentiated thyroid carcinoma(UTC), and medullary carcinoma (MTC) [4], and there have been different mutational profiles in these subtypes. Recent studies have confirmed a few molecular markers that have allowed better understanding of the molecular mechanisms of TC, for instance, BRAF and RAS point mutations, RET/PTC and PAX8/PPAR gene rearrangements, etc [5–7]. In addition, the latest study found that Substance P/neurokinin-1 receptor (SP/NK-1R) system was over-expressed in TC than that in normal thyroid tissues via immunohistochemical study, which could promote the migration and invasion of cancer cells [8]. Targeting certain molecules associated with initiation and progression of TC will be a significant and promising research area. Therefore, it was critical to continue to study the molecular mechanisms involved in TC, which may provide new strategies for the diagnosis and treatment of TC patients in the future.

Basigin (also known as BSG, CD147, or Extracellular Matrix Metalloproteinase inducer [EMMPRIN]) is a single pass type 1 transmembrane protein that plays a crucial part in developmental processes, wound healing, nutrient transport, inflammation, arthritis, and microbial pathologies. BSG has also been verified as a potential stimulator of matrix metalloproteinases (MMPs) and is considered to be a prognostic marker in cancer [9]. Accumulating studies have found that BSG was over-expressed in many different human tumor cell types, such as those caused by brain cancer, colon cancer, cervical cancer, and endometrial cancer. It also played a crucial role in tumor cell invasion, metastasis, and angiogenesis [10–14]. The role of BSG in cancer suggests that it could be an effective therapeutic target. For instance, exciting clinical progress has been made in hepatocellular carcinoma (HCC) treatment by means of BSG-directed monoclonal antibodies [15].

As for TC, BSG also played an important role in the tumorigenicity, invasion, metastasis, and degree of dedifferentiation [16, 17]. Recently, researchers have identified that BSG expression regulates tumor cell glycolysis, resulting in the progression of TC [17, 18]. In addition, researchers have also found that BSG gene silencing leads to growth inhibition of thyroid medullary carcinoma TT cells and alteration of the cell cycle [17]. Moreover, it was proved that BSG was involved in the invasiveness of FTC cells via regulation of MMPs [19]. For differentiated carcinoma (DTC), the expression of BSG and MMP-2 may be an important feature, and the expression of BSG may be useful to predict the prognosis of DTC patients [20, 21]. Furthermore, a study demonstrated that BSG inhibition may be a therapeutic target for TC patients [18].

In summary, accumulating evidence suggested that BSG exerts a variety of functions in TC progression. However, until now, only one study mentioned a possible molecular mechanism underlying TC: miR-125a-5p regulated BSG and was negatively correlated with its expression and function [18]. Within this context, little was known about the molecular mechanism of BSG in TC, and it was essential to further investigate its expression and function.

In this study, we analyzed cases gathered from immunohistochemistry (IHC), RNA-sequencing (RNA-seq), and gene microarray data to provide evidence to attest to the clinical value of BSG in TC. Co-expressed and differentially expressed genes (DEGs) of BSG in TC, along with the corresponding intersection genes and hub genes, were also obtained. In addition, Gene Ontology (GO) and Kyoto Encyclopedia of Genes and Genomes (KEGG) enrichment analyses were performed to intersect genes and explore the potential mechanisms of BSG in TC. We hope that our findings will broaden the horizon for future studies of TC and BSG.

## Materials and methods

### BSG expression level in TC

#### Detecting protein expression levels of BSG by in-house immunohistochemistry

Two tissue microarrays (THC961 and THC1021) which contained 23 non-cancerous thyroid tissues and 125 TC (including PTC, FTC, MTC, and ATC) tissues were obtained from Fanpu Biotech, Inc. (Guilin, China). In addition, a number of 64 non-cancerous thyroid tissues and 46 TC tissues were collected from the Department of Pathology, First Affiliated Hospital of Guangxi Medical University, from March 1 to December 1, 2018. The study was approved by the Ethical Committee of the First Affiliated Hospital of Guangxi Medical University. And all patients provided written informed consents for use of their samples in study. Subsequently, pathologists (Wei-jia Mo and Gang Chen) independently evaluated all slides using a semi-quantitative scoring system without knowing the clinical results in advance to classify the staining intensity and the percentage of positive cancer cells. The staining intensity score was divided into 0 (negative), 1 (weak), 2 (medium), or 3 (strong). And the proportion of staining score set as 0 (< 10%), 1 (11–25%), 2 (26–50%), 3 (51–75%), or 4 (76–100%). Finally, the final immune response score was determined by combining the intensity and proportion scores. In addition, a correlation analysis was constructed to determine the relationships between BSG expression levels, clinicopathological characteristics, and patient prognosis. Furthermore, we supplemented the immunohistochemical results of the BSG expression levels in thyroid cancer by searching the available literature and The Human Protein Atlas [22].

#### BSG mRNA expression in TC samples from TCGA RNA-seq data

Using The Cancer Genome Atlas (TCGA) database, we downloaded BSG expression data related to TC and para-cancerous tissues [23]. Then, the downloaded data were integrated and transferred into the quantile normalized log2 format to improve measurement accuracy. SPSS 25.0 (SPSS Inc. Chicago, IL, USA) was used to calculate the mean BSG expression and standard deviation (SD) in 505 TC tissues and 59 non-cancer tissues, incorporating clinical information gathered from the TCGA database. Furthermore, a Kaplan-Meier survival curve combined with TC patients’ survival data was constructed using GraphPad Prism Version 7.0, in order to estimate the prognostic capacity of BSG in TC.

#### BSG expression in TC tissues from other microarray data

This study included The Gene Expression Database (GEO) [24], ArrayExpress [25], Oncomine [26], and The Sequence Read Archive (SRA) [27] in order to search both Chinese and English medical literature for microarray data of BSG to analyze its clinical value in TC. Our search included data entered into the databases from the beginning to February 21, 2020. The retrieval formula of this study was as follows: (cancer OR carcinoma OR adenocarcinoma OR tumour OR tumor OR malignanc* OR neoplas*) AND (Thyroid OR Thyroidea OR Glandula thyroidea) AND (mRNA OR gene OR “messenger RNA”). Finally, only microchip data relevant to human samples that included BSG expression in TC tissues and non-cancerous tissues were selected.

#### Searching the literature for BSG expression

In order to supplement the new data on BSG expression in recent studies, we conducted a comprehensive literature search of electronic databases (including PubMed, Embase, Web of Science, Wiley Online Library, SpringerLink, Chinese National Knowledge Infrastructure, Chinese Biomedical Literature Database, Chinese VIP, and Wanfang database) to obtain eligible studies. The search retrieval formula for these databases was as follows: (cancer OR carcinoma OR adenocarcinoma OR tumour OR tumor OR malignanc* OR neoplas*) AND (Thyroid OR Thyroidea OR Glandula thyroidea) AND (“Basigin” OR “Ok blood group” OR “CD147” OR “EMMPRIN”). Only studies that included human BSG expression levels were selected.

#### Comprehensive analysis of BSG expression in TC

Data from four types of sources (IHC, RNA-seq, microarrays, and literature) were combined using Stata Version 12.0 in order to perform a comprehensive analysis to determine BSG expression levels in TC, and the standard mean difference (SMD) and 95% confidence interval (CI) were calculated. Furthermore, the summary receiver operating characteristic (sROC) curve was used to distinguish TC tissues from non-cancerous tissues, which were gathered from the above databases. Sensitivity and specificity, as well as the corresponding likelihood ratio (LR) and diagnostic odds ratio (DOR) were calculated using meta-disk Version 1.4 [28–30].

### Validation of the expression of BSG using cell line data from CCLE

A search for the term “BSG” was performed on the Cancer Cell Line Encyclopedia (CCLE) database [31], and a heat-map based on the expression of BSG in different TC cell lines was constructed using HemI (Heatmap Illustrator, version 1.0).

### The potential mechanism of BSG expression in thyroid cancer

#### Identification of BSG co-expressed genes and differentially expressed genes (DEGs) in TC

To obtain the hub genes of BSG in TC, we first searched the cBioPortal for Cancer Genomics [32] and found the co-expressed genes of BSG. The DEGs from all gene microarray datasets and RNA-seq data were obtained using the RRA method. This method uses a probabilistic model for aggregation, which is resilient to noise, and can calculate the probability of significance of all elements and perform the final ranking [33–37]. The cutoff *P* value for the RRA was set as 0.05.

Eventually, the genes that intersected with the co-expressed genes and DEGs were selected for subsequent work.

#### GO and KEGG clustering analyses of BSG co-expressed genes and DEGs in TC

The BSG co-expressed genes and DEGs in TC were uploaded into a function annotation portal via The Database for Annotation, Visualization, and Integrated Discovery (DAVID) [38], an R studio software that provides a systematic and comprehensive analysis for gene lists.

#### Identification of BSG hub genes in TC

The protein interactions of BSG co-expressed genes and DEGs were obtained using the Search Tool for the Retrieval of Interacting Genes (STRING) network database, and we set the minimum required interaction score at 0.7. We then used CytoHubba, a plug-in of Cytoscape, which can identify hub objects and sub-networks from complex interactome to calculate gene nodes present in the interaction network. We imported PPI results of STRING website into Cytoscape, selected the CytoHubba plug-in, and calculated the top ten genes through MCC algorithm which has a better performance on the precision of predicting essential proteins from the yeast PPI network [39]. And the network interface will show the connection of these hub nodes in the network. The darker the node color, the higher the score. Ultimately, the top 10 genes were selected as BSG hub genes in TC.

### Statistical analysis

SPSS 25.0 software was chosen for data analysis, which included an independent Student’s *t* test to determine the significance of BSG expression in two groups and a univariate analysis for three or more groups. A *P* value below 0.05 was considered statistically significant. Scatter diagrams whose data ranged between normal and TC tissues were prepared by the GraphPad Prism 7.0 software. In addition, we established a ROC curve to evaluate the diagnostic role of BSG in TC and differentiated TC and non-cancerous tissue by area under the curve (AUC), which can be used as a diagnostic. Moreover, according to the AUC, the diagnostic efficacy can be divided into three levels: low (0.5–0.7), medium (0.7–0.9), and high (0.9–1.0).

## Results

### Immunohistochemical investigation for BSG protein expression levels

**Fig. 1:**
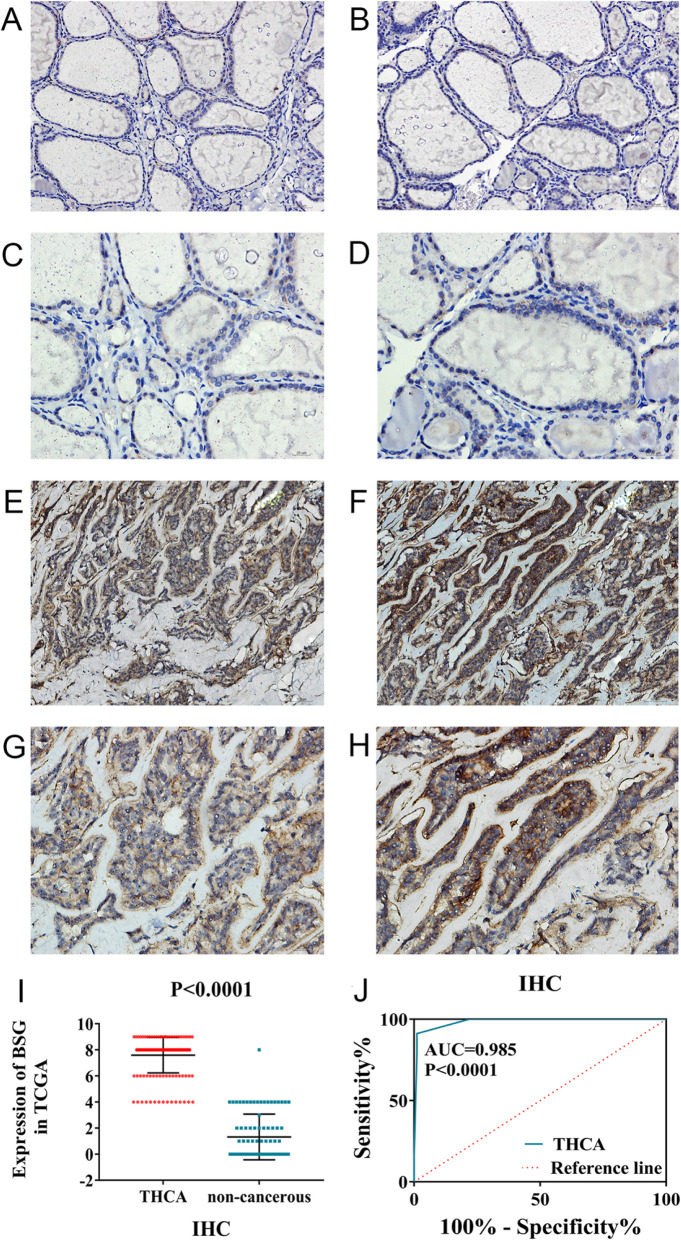
The results of BSG immunohistochemical staining. **a** BSG staining in non-cancer tissues (× 200). **b** BSG staining in non-cancer tissues (× 200). **c** BSG staining in non-cancer tissues (× 400). **d** BSG staining in non-cancer tissues (× 400). **e** BSG staining in TC tissues (× 200). **f** BSG staining in TC tissues (× 200). **g** BSG staining in TC tissues (× 400). **h** BSG staining in TC tissues (× 400). **i** The expression of BSG in 171 TC and 87 non-cancerous thyroid tissues. **j** The ROC curve was generated to assess the diagnostic ability of BSG in 171 TC and 87 non-cancerous thyroid tissues. The AUC was 0.985 (95%CI 0.971 to 0.999, *p* < 0.0001), and the corresponding sensitivity and specificity was 0.912 and 0.966, respectively

This study included 171 cases of TC tissues and 87 cases of non-cancerous tissues, which were collected from the First Affiliated Hospital of Guangxi Medical University (Fig. 1a–h). Based on these samples, the BSG protein expression in the 171 cases of TC tissues was notably higher than that in the 87 cases of non-cancerous tissues (7.6 ± 1.366 vs. 1.32 ± 1.749, *p* < 0.0001) (Fig. 1i). The AUC of BSG protein expression used to distinguish between TC cases and non-cancerous cases was 0.985 (95%CI 0.971 to 0.999, *p* < 0.0001). The sensitivity and specificity were 0.912 and 0.966, respectively (Fig. 1j). In addition, the relationship between BSG expression and clinical parameters was shown supplementary Table S1.

### BSG mRNA expression in TC samples

**Fig. 2:**
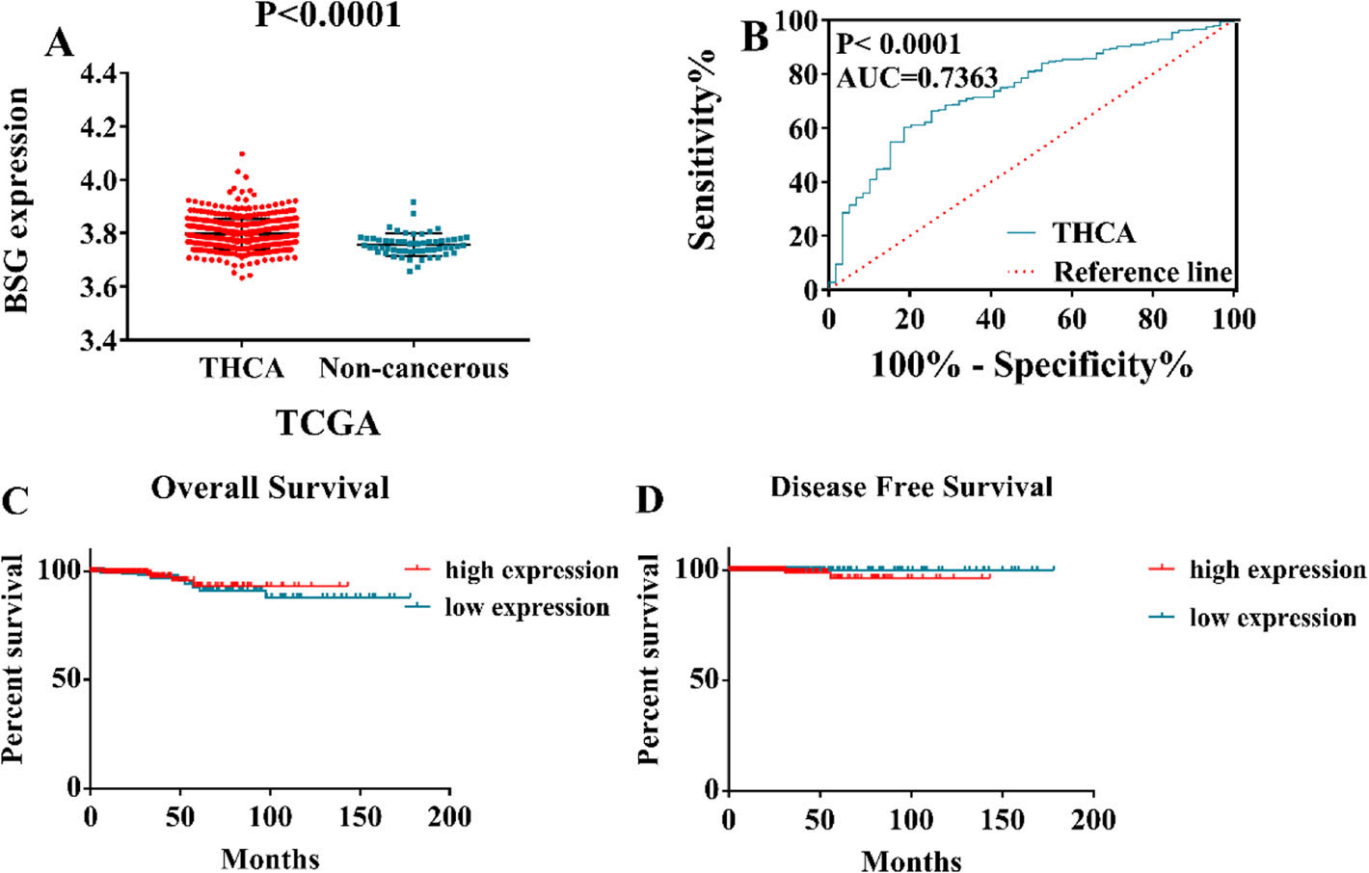
Clinical role of upregulation of BSG mRNA level in thyroid cancer (TC) based on RNA-sequencing data. **a**, **b** Expression level of BSG mRNA between TC and non-cancerous thyroid controls. **c** Overall survival, log-rank *p* = 0.5027, hazard ratio (log-rank) = 0.6988 (95%CI 0.2564 to 1.905). **d** Disease-free survival, log-rank *p* = 0.0947, hazard ratio (Mantel-Haenszel) = 10.98 (95%CI 0.6606 to 182.5)

In total, we collected 505 TC samples and 59 non-cancerous samples for RNA-seq data analysis. Corresponding clinical information was also collected and was shown in Table 1. The BSG mRNA expression level in the TC samples was dramatically higher than that in the non-cancerous samples (3.8 ± 0.056 vs. 3.76 ± 0.042, *p* < 0.0001). Furthermore, the AUC of BSG mRNA expression was 0.7363 (95%CI 0.6743 to 0.7983, *p* < 0.0001), and the sensitivity and specificity were 0.602 and 0.814, respectively (Fig. 2a, b). Subsequently, the prognostic value of BSG expression was inspected. The Kaplan-Meier survival curve analysis revealed that TC patients with low BSG expression levels were more likely to have worse overall survival rates (Fig. 2c, d) than those with high BSG expression levels (log-rank *p* = 0.5027). In addition, the HR value was 0.6988 (95%CI 0.2564 to 1.905). More interestingly, TC patients with high BSG expression levels tended to have worse rates of disease-free survival than those with low BSG expression levels (log-rank *p* = 0.0947), and the HR value was 10.98 (95%CI 0.6606 to 182.5).

**Table 1:** Clinical pathological parameters and BSG expression in TC data from TCGA database

Characteristic		Expression of BSG (log_2_*x*)			
		*n*	Mean ± SD	*t*/*F* value	*p* value
Tissue	TC	505	3.8 ± 0.056	6.647	**< 0.0001**
	Non-cancerous	59	3.76 ± 0.042		
Gender	Female	369	3.7946 ± 0.05313	1.175	0.081
	Male	136	3.8044 ± 0.06241		
Age	≥ 60	120	3.8031 ± 0.05706	− 1.33	0.184
	< 60	385	3.7954 ± 0.05546		
Pathologic T	T1	143	3.7887 ± 0.05514	1.5	0.201
	T2	166	3.7991 ± 0.05678		
	T3	171	3.8006 ± 0.05538		
	T4	23	3.8128 ± 0.04993		
	Tx	2	3.7882 ± 0.12413		
T	T1–T2	309	3.7943 ± 0.05618	1.17	0.311
	T3–T4	194	3.802 ± 0.05479		
	Tx	2	3.7882 ± 0.12413		
Pathologic N	N0	230	3.8047 ± 0.06035	4.387	**0.013**
	N1	225	3.7893 ± 0.04527		
	Nx	50	3.7985 ± 0.07198		
Pathologic M	M0	283	3.794 ± 0.05303	1.922	0.147
	M1	9	3.8248 ± 0.05971		
	Mx	213	3.8004 ± 0.05911		
Stage	I	285	3.7925 ± 0.05688	4.011	**0.008**
	II	52	3.8195 ± 0.06654		
	III	113	3.7948 ± 0.04918		
	IV	55	3.8057 ± 0.04748		
Stage	I–II	337	3.7967 ± 0.05918	− 0.323	0.747
	III–IV	168	3.7984 ± 0.04876		
Subtype	Other, specify	9	3.8297 ± 0.06582	14.593	**< 0.0001**
	Thyroid papillary carcinoma—classical/usual	358	3.7883 ± 0.04898		
	Thyroid papillary carcinoma—follicular	102	3.8266 ± 0.06996		
	Thyroid papillary carcinoma—tall cell	36	3.7948 ± 0.0421		

*TC* thyroid cancer

### BSG expression in TC tissues according to the microarray data analysis

**Fig. 3:**
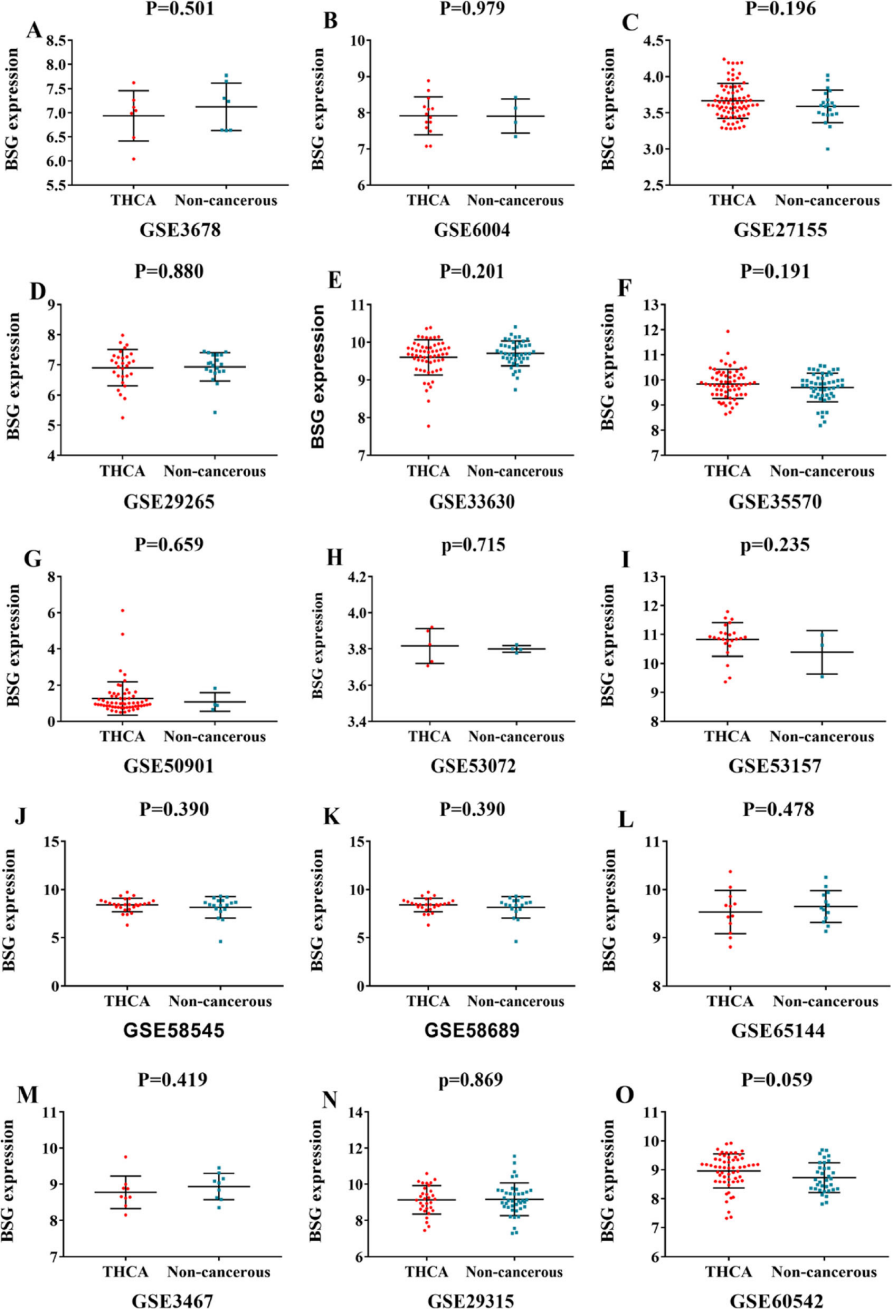
Scatter plots of BSG mRNA expression in thyroid cancer (TC) based on 15 GEO gene microarrays. **a** Microarray GSE3678. **b** MicroarrayGSE6004. **c** Microarray GSE27155. **d** Microarray GSE29265. **e** Microarray GSE33630. **f** Microarray GSE35570. **g** Microarray GSE50901. **h** Microarray GSE53072. **i** Microarray GSE53751. **j** Microarray GSE58545. **k** Microarray GSE58689. **l** Microarray GSE65144. **m** Microarray GSE3467. **n** Microarray GSE29315. **o** Microarray GSE60542

After the retrieval using the GEO database, 15 microarrays of BSG expression datasets were selected for our study. These microarrays contained 506 TC cases and 291 non-cancerous cases (Fig. 3, Table 2). Of these 15 microarrays, 9 (GSE27155, GSE53072, GSE6004, GSE58545, GSE35570, GSE53157, GSE50901, GSE60542, and GSE58689) showed that the expression levels of BSG in TC cases were slightly higher than that in non-cancerous cases. In order to further analyze the BSG expression levels in these microarrays, we merged the microarray data and performed a meta-analysis. The combined SMD for BSG was 0.11 (95%CI − 0.04 to 0.25) based on the fixed effect model (Supplementary Figure S1A). The *I*-squared value and the *P* value were 0.0% and 0.570, respectively. A sensitivity analysis of BSG expression levels in TC tissues and non-cancerous tissues was performed to show the differences among the microarrays (Supplementary Figure S1B). In addition, the publication bias was assessed by means of a funnel plot (Supplementary Figures S1C and S1D). The publication bias was not significant for this meta-analysis of BSG expression according to Begg’s test (*p* = 0.805) and Egger’s test (*p* = 0.745). Furthermore, the funnel plot also displayed a symmetric shape.

**Table 2:** Expression levels of *BSG* in the microarrays

ID	Year		*n*	Mean ± SD	*t* value	*P* value
GSE27155	2011	TC	78	3.6650 ± 0.2420	1.302	0.196
		Non-cancerous	21	3.5886 ± 0.2249		
GSE53072	2013	TC	5	3.8166 ± 0.0956	0.389	0.715
		Non-cancerous	4	3.7995 ± 0.0188		
GSE65144	2015	TC	12	9.5326 ± 0.4483	− 0.722	0.478
		Non-cancerous	13	9.6457 ± 0.3306		
GSE29315	2012	TC	31	9.1377 ± 0.7861	− 0.166	0.869
		Non-cancerous	40	9.1715 ± 0.9021		
GSE3678	2006	TC	7	6.9376 ± 0.251	− 0.693	0.501
		Non-cancerous	7	7.1255 ± 0.4929		
GSE6004	2006	TC	14	7.9174 ± 0.5241	0.027	0.979
		Non-cancerous	4	7.9096 ± 0.4714		
GSE29265	2012	TC	29	6.9111 ± 0.5993	− 0.151	0.880
		Non-cancerous	20	6.9352 ± 0.4666		
GSE58545	2015	TC	27	8.3943 ± 0.7030	0.868	0.390
		Non-cancerous	18	8.1597 ± 1.1135		
GSE58689	2015	TC	27	8.3943 ± 0.7030	0.868	0.390
		Non-cancerous	18	8.1597 ± 1.1135		
GSE35570	2015	TC	65	9.8489 ± 0.5833	1.315	0.191
		Non-cancerous	51	9.7066 ± 0.5724		
GSE33630	2012	TC	59	9.6007 ± 0.4657	− 1.286	0.201
		Non-cancerous	45	9.7057 ± 0.3300		
GSE53157	2013	TC	24	10.8278 ± 0.5792	1.218	0.235
		Non-cancerous	3	10.3846 ± 0.7451		
GSE50901	2014	TC	61	1.3010 ± 0.8936	0.444	0.659
		Non-cancerous	4	1.0997 ± 0.4919		
GSE3467	2005	TC	9	8.7789 ± 0.4506	− 0.829	0.419
		Non-cancerous	9	8.9390 ± 0.3642		
GSE60542	2015	TC	58	8.9624 ± 0.5898	1.910	0.059
		Non-cancerous	34	8.7006 ± 0.5161		

The combined sensitivity of BSG was 0.47 (95%CI 0.42 to 0.51, *p* < 0.0001), and the specificity was 0.74 (95%CI 0.69 to 0.79, *p* < 0.0001) with a positive likelihood ratio of 1.53 (95%CI 1.26 to 1.86, *p* = 0.9913), a negative likelihood ratio of 0.79 (95%CI 0.69 to 0.90, *p* = 0.0367), and a diagnostic odds ratio of 2.38 (95%CI 1.65 to 3.43, *p* = 0.9277). Most importantly, the AUC of BSG reached 0.6539, showing that BSG has a good capacity to differentiate TC tissues from the non-cancerous tissues (Supplementary Figure S2).

### Integrated analysis of the in-house immunohistochemistry, RNA-seq, microarray, and BSG expression data reported in the literature

**Fig. 4:**
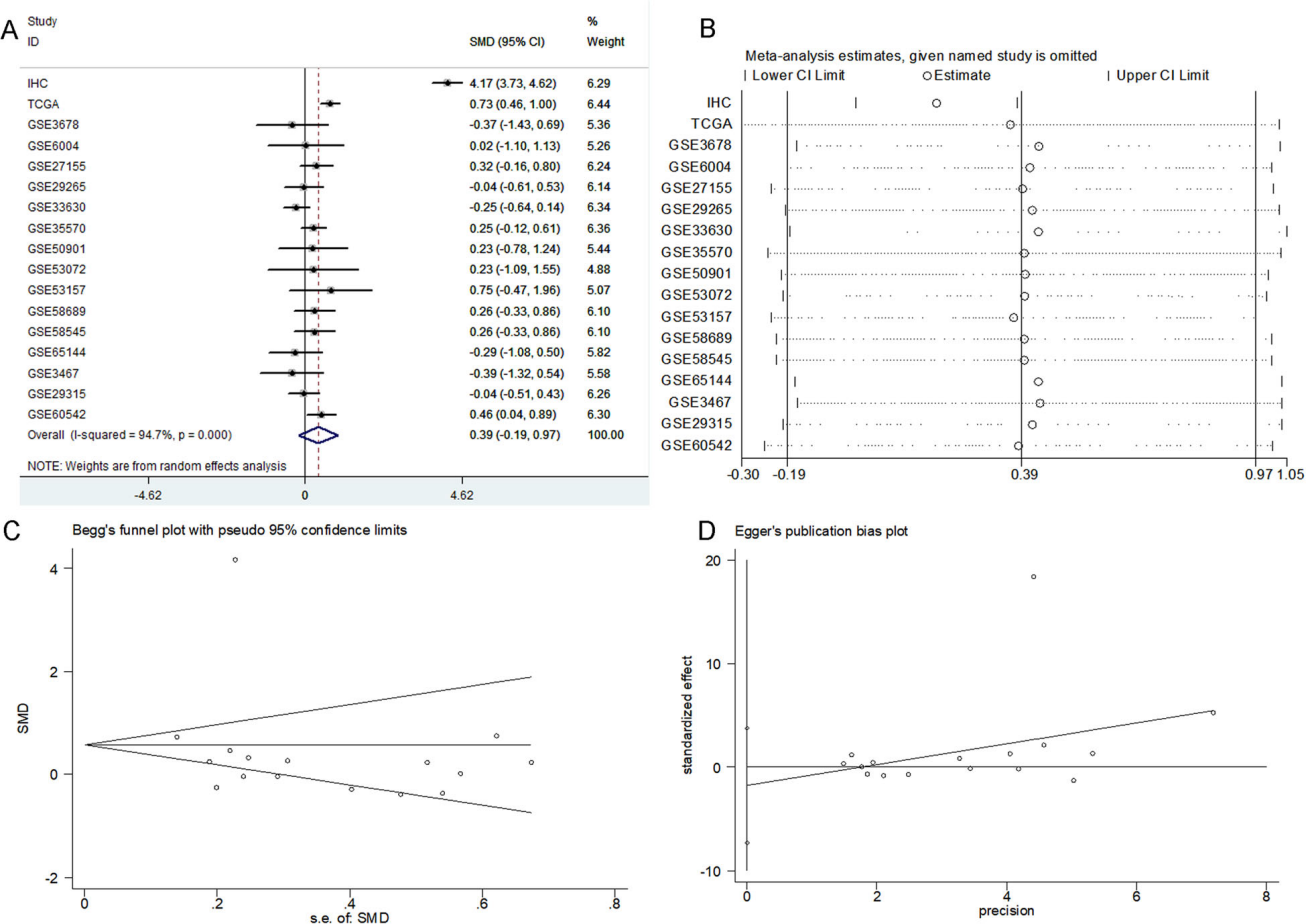
Integrated assessment of the BSG mRNA level in thyroid cancer (TC) based on all available data. **a** Forest plot. **b** Sensitivity. **c** Funnel plot (Begg’s test). **d** Funnel plot (Egger’s test)

**Fig. 5:**
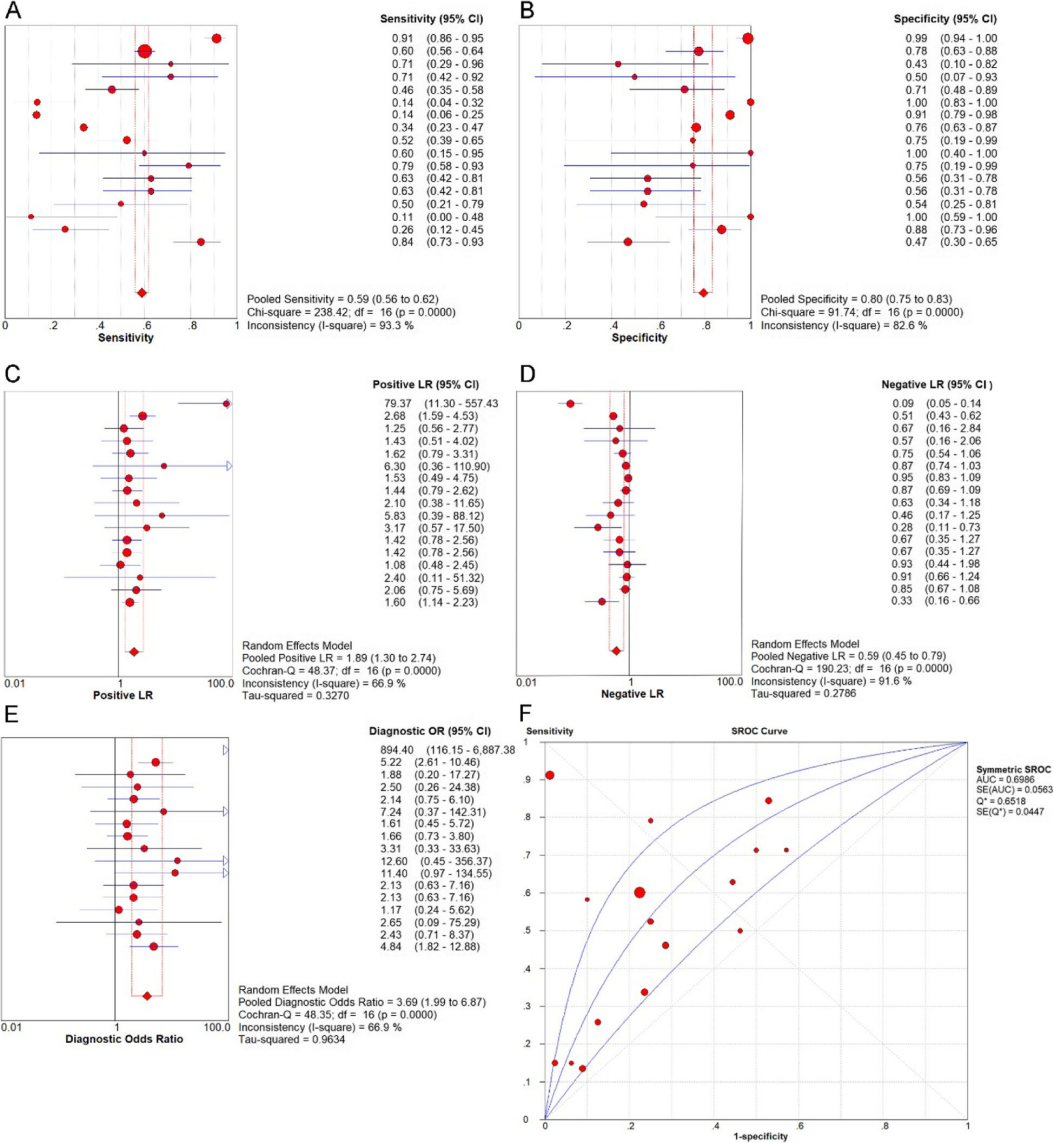
Expression level of BSG confirmed using sROC curves including all available data. **a** Sensitivity. **b** Specificity. **c** Positive likelihood ratios. **d** Negative likelihood ratios. **e** Diagnostic odds ratio. **f** AUC of sROC

In order to comprehensively and systematically analyze the BSG expression level in TC, we performed a meta-analysis combining all sources of data, which included 1182 TC cases and 437 non-cancerous cases (*n* = 17). The combined SMD for BSG expression was 0.39 (95%CI − 0.19 to 0.97) according to a random effect model (*I*-squared value, 94.7%). In addition, the *P* value was < 0.0001, confirming a significant difference in the sensitivity analysis (Fig. 4a, b). Moreover, the publication bias was not significant according to Begg’s test (*p* = 0.323) and Egger’s test (*p* = 0.508) (Fig. 4c, d). The combined sensitivity of BSG was 0.59 (95%CI 0.56 to 0.62, *p* < 0.0001), and the specificity was 0.80 (95%CI 0.75 to 0.83, *p* < 0.0001) with a positive likelihood ratio of 1.89 (95%CI 1.30 to 2.74, *p* < 0.0001), a negative likelihood ratio of 0.59 (95%CI 0.45 to 0.79, *p* < 0.0001), and a diagnostic odds ratio of 3.69 (95%CI 1.99 to 6.87, *p* < 0.0001). Ultimately, the AUC of the sROC curve, which estimated the capacity of BSG expression to distinguish TC cases from non-cancerous cases, was 0.6986 (Fig. 5).

### Validation of BSG expression using CCLE cell coefficient data

**Fig. 6:**
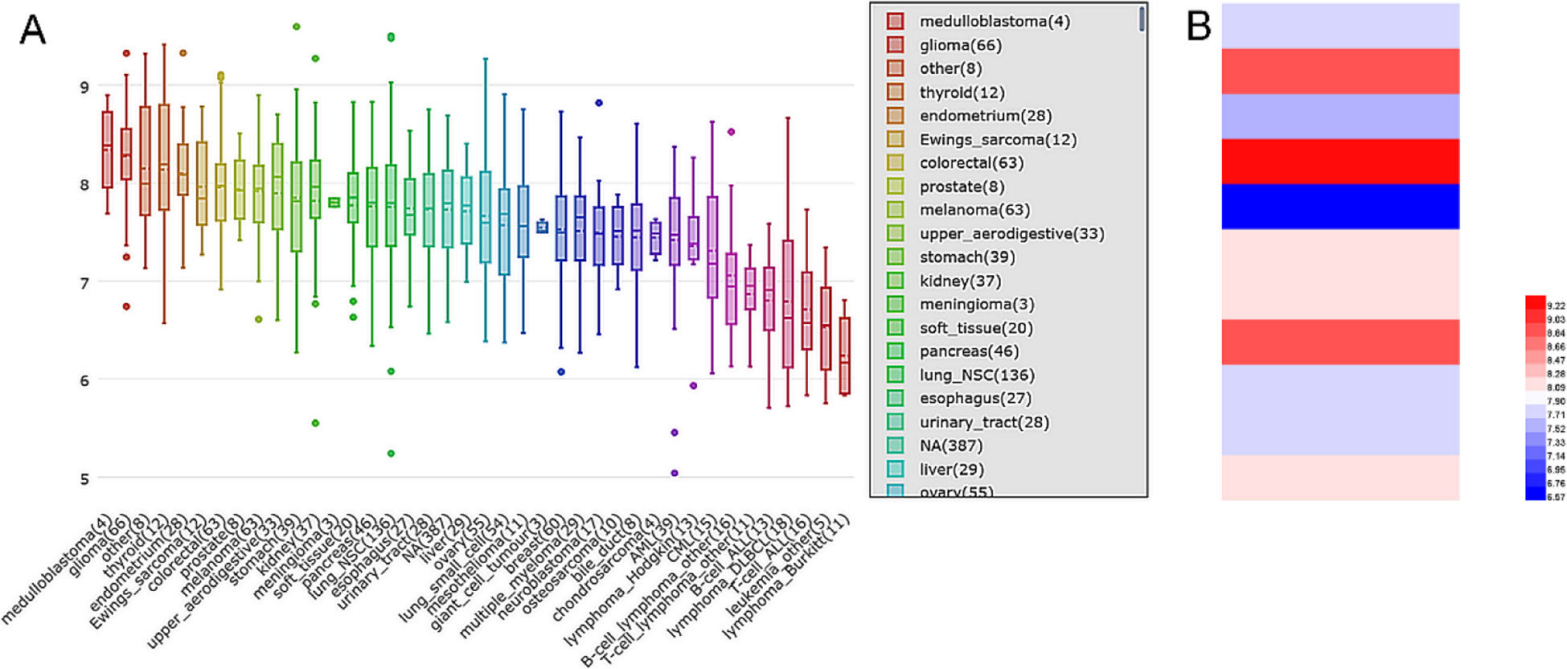
Expression of BSG mRNA in thyroid cancer (TC) cell lines from the Cancer Cell Line Encyclopedia (CCLE). **a** Expression of BSG mRNA in all cell types. **b** Expression of BSG mRNA in all TC cell lines

Using the CCLE database, we found that the expression levels of BSG in thyroid cell lines were higher than that in the majority of other types of cell lines (Fig. 6a). A heat-map of BSG expression in the TC cell lines was also constructed, and over half of the bands were red (Fig. 6b).

### The potential functions and pathways of BSG in TC

**Fig. 7:**
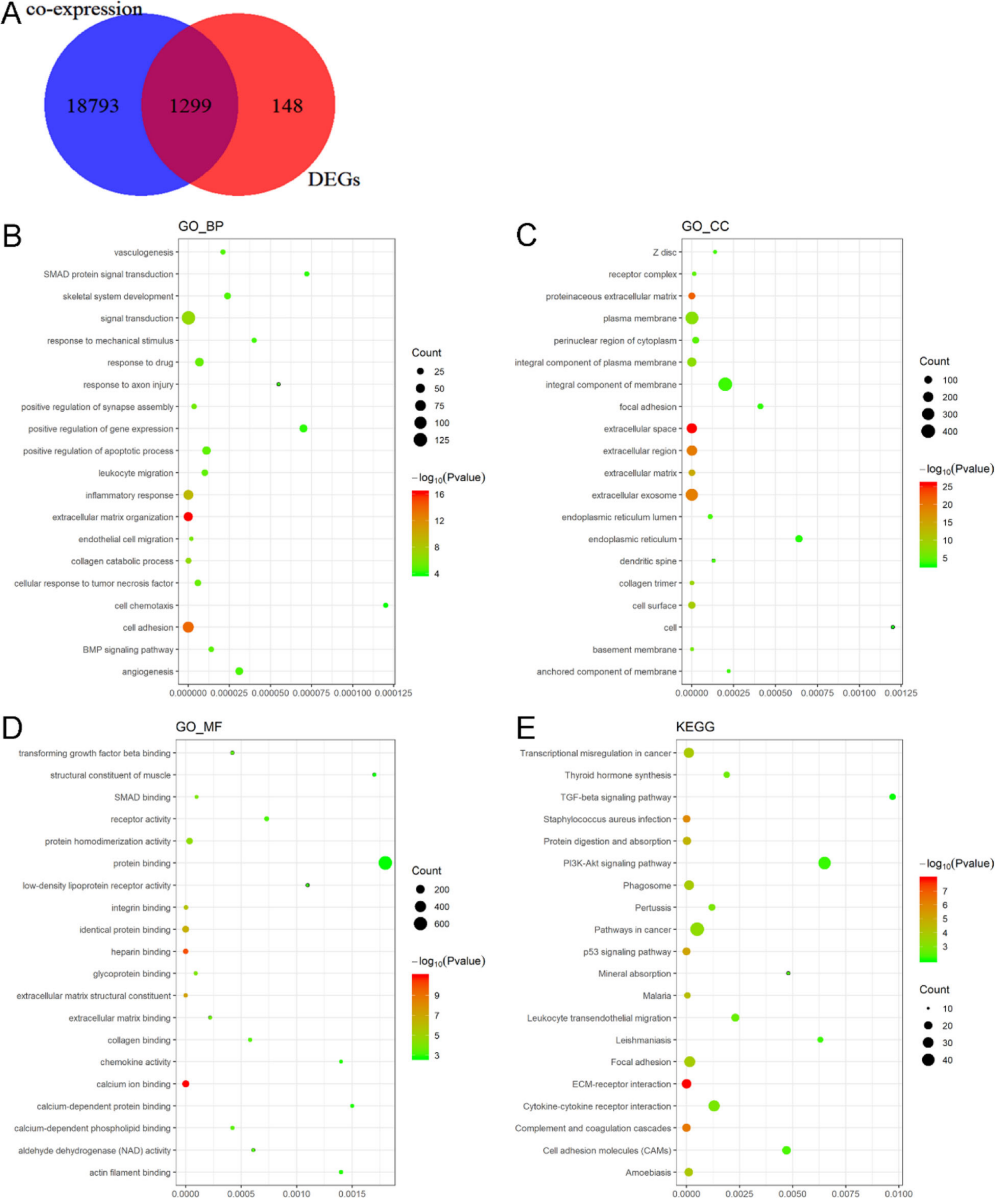
The potential mechanism of BSG in thyroid cancer (TC) assessed by GO annotation and KEGG enrichment analysis. **a** Interaction of BSG co-expressed genes and differentially expressed genes (DEGs) in TC. **b–d** GO analyses. **e** KEGG enrichment analysis

We discovered 20092 BSG co-expressed genes using the cBioPortal for Cancer Genomics and 1447 DEGs using RRA methods. Finally, we discovered 1299 BSG co-expressed genes and DEGs by performing an intersection between the co-expressed gene data and the DEG data (Fig. 7a).

Afterwards, GO and KEGG analyses were performed on these 1299 genes. The GO enrichment analysis unveiled enrichment of extracellular matrix (ECM) organization, cell adhesion, and inflammatory response in biological processes (BP). And we also discovered enrichment of extracellular space, proteinaceous extracellular matrix, and extracellular region in cellular component (CC). Furthermore, enrichment of calcium ion binding, heparin binding and extracellular matrix structural constituent were also found in molecular function (MF) (Fig. 7b–d, Table 3). Most importantly, the result of the KEGG analysis revealed that co-expressed genes and DEGs of BSG tended to enrich in the following terms: ECM-receptor interactions, complement and coagulation cascades, *Staphylococcus aureus* infection, p53 signaling pathway, and protein digestion and absorption (Fig. 7e, Table 3).

**Table 3:** Enrichment of functions and signaling pathways with BSG-related gene in thyroid cancer

Category	Term	Count	%	*P* value
GO_BP	Extracellular matrix organization	52	4	5.9E− 17
GO_BP	Cell adhesion	81	6.2	1.4E− 14
GO_BP	Inflammatory response	62	4.8	6E− 10
GO_BP	Signal transduction	128	9.8	1.5E− 07
GO_BP	Collagen catabolic process	19	1.5	2.2E− 07
GO_BP	Endothelial cell migration	12	0.9	1.9E− 06
GO_BP	Positive regulation of synapse assembly	17	1.3	3.5E− 06
GO_BP	Cellular response to tumor necrosis factor	23	1.8	5.8E− 06
GO_BP	Response to drug	44	3.4	6.9E− 06
GO_BP	Leukocyte migration	24	1.8	0.00001
GO_BP	Positive regulation of apoptotic process	43	3.3	0.000011
GO_BP	BMP signaling pathway	18	1.4	0.000014
GO_BP	Vasculogenesis	15	1.2	0.000021
GO_BP	Skeletal system development	25	1.9	0.000024
GO_BP	Angiogenesis	34	2.6	0.000031
GO_BP	Response to mechanical stimulus	15	1.2	0.00004
GO_BP	Response to axon injury	10	0.8	0.000055
GO_BP	Positive regulation of gene expression	37	2.8	0.00007
GO_BP	SMAD protein signal transduction	15	1.2	0.000072
GO_BP	Cell chemotaxis	15	1.2	0.00012
GO_CC	Extracellular space	199	15.3	1.9E− 26
GO_CC	Proteinaceous extracellular matrix	70	5.4	1.3E− 22
GO_CC	Extracellular region	208	16	2.4E− 20
GO_CC	Extracellular exosome	311	23.9	6E− 20
GO_CC	Extracellular matrix	62	4.8	4.5E− 15
GO_CC	Cell surface	80	6.2	1E− 10
GO_CC	Collagen trimer	26	2	1.5E− 09
GO_CC	Integral component of plasma membrane	151	11.6	2.5E− 08
GO_CC	Plasma membrane	361	27.8	4.4E− 08
GO_CC	Basement membrane	20	1.5	1.1E− 06
GO_CC	Receptor complex	24	1.8	0.000015
GO_CC	Perinuclear region of cytoplasm	71	5.5	0.000023
GO_CC	Endoplasmic reticulum lumen	29	2.2	0.00011
GO_CC	Dendritic spine	19	1.5	0.00013
GO_CC	Z disk	21	1.6	0.00014
GO_CC	Integral component of membrane	409	31.5	0.0002
GO_CC	Anchored component of membrane	20	1.5	0.00022
GO_CC	Focal adhesion	46	3.5	0.00041
GO_CC	Endoplasmic reticulum	82	6.3	0.00064
GO_CC	Cell	17	1.3	0.0012
GO_MF	Calcium ion binding	99	7.6	1.2E− 11
GO_MF	Heparin binding	37	2.8	1E− 10
GO_MF	Extracellular matrix structural constituent	20	1.5	5.3E− 08
GO_MF	Identical protein binding	89	6.8	2.1E− 07
GO_MF	Integrin binding	23	1.8	1.6E− 06
GO_MF	Protein homodimerization activity	79	6.1	0.000035
GO_MF	Glycoprotein binding	15	1.2	0.000089
GO_MF	SMAD binding	12	0.9	0.000098
GO_MF	Extracellular matrix binding	9	0.7	0.00022
GO_MF	Transforming growth factor beta binding	7	0.5	0.00042
GO_MF	Calcium-dependent phospholipid binding	13	1	0.00042
GO_MF	Collagen binding	13	1	0.00058
GO_MF	Aldehyde dehydrogenase (NAD) activity	7	0.5	0.00061
GO_MF	Receptor activity	29	2.2	0.00073
GO_MF	Low-density lipoprotein receptor activity	6	0.5	0.0011
GO_MF	Chemokine activity	11	0.8	0.0014
GO_MF	Actin filament binding	20	1.5	0.0014
GO_MF	Calcium-dependent protein binding	12	0.9	0.0015
GO_MF	Structural constituent of muscle	10	0.8	0.0017
GO_MF	Protein binding	642	49.4	0.0018
KEGG_PATHWAY	ECM-receptor interaction	25	1.9	1.3E− 08
KEGG_PATHWAY	Complement and coagulation cascades	20	1.5	4.5E− 07
KEGG_PATHWAY	*Staphylococcus aureus* infection	17	1.3	1.2E− 06
KEGG_PATHWAY	p53 signaling pathway	18	1.4	6.2E− 06
KEGG_PATHWAY	Protein digestion and absorption	20	1.5	0.000022
KEGG_PATHWAY	Malaria	14	1.1	0.000046
KEGG_PATHWAY	Amoebiasis	21	1.6	0.0001
KEGG_PATHWAY	Transcriptional misregulation in cancer	28	2.2	0.00012
KEGG_PATHWAY	Phagosome	26	2	0.00013
KEGG_PATHWAY	Focal adhesion	32	2.5	0.00015
KEGG_PATHWAY	Pathways in cancer	49	3.8	0.0005
KEGG_PATHWAY	Pertussis	15	1.2	0.0012
KEGG_PATHWAY	Cytokine-cytokine receptor interaction	33	2.5	0.0013
KEGG_PATHWAY	Thyroid hormone synthesis	14	1.1	0.0019
KEGG_PATHWAY	Leukocyte transendothelial migration	19	1.5	0.0023
KEGG_PATHWAY	Cell adhesion molecules (CAMs)	21	1.6	0.0047
KEGG_PATHWAY	Mineral absorption	10	0.8	0.0048
KEGG_PATHWAY	Leishmaniasis	13	1	0.0063
KEGG_PATHWAY	PI3K-Akt signaling pathway	40	3.1	0.0065
KEGG_PATHWAY	TGF-beta signaling pathway	14	1.1	0.0097

### Hub genes of BSG in TC

**Fig. 8:**
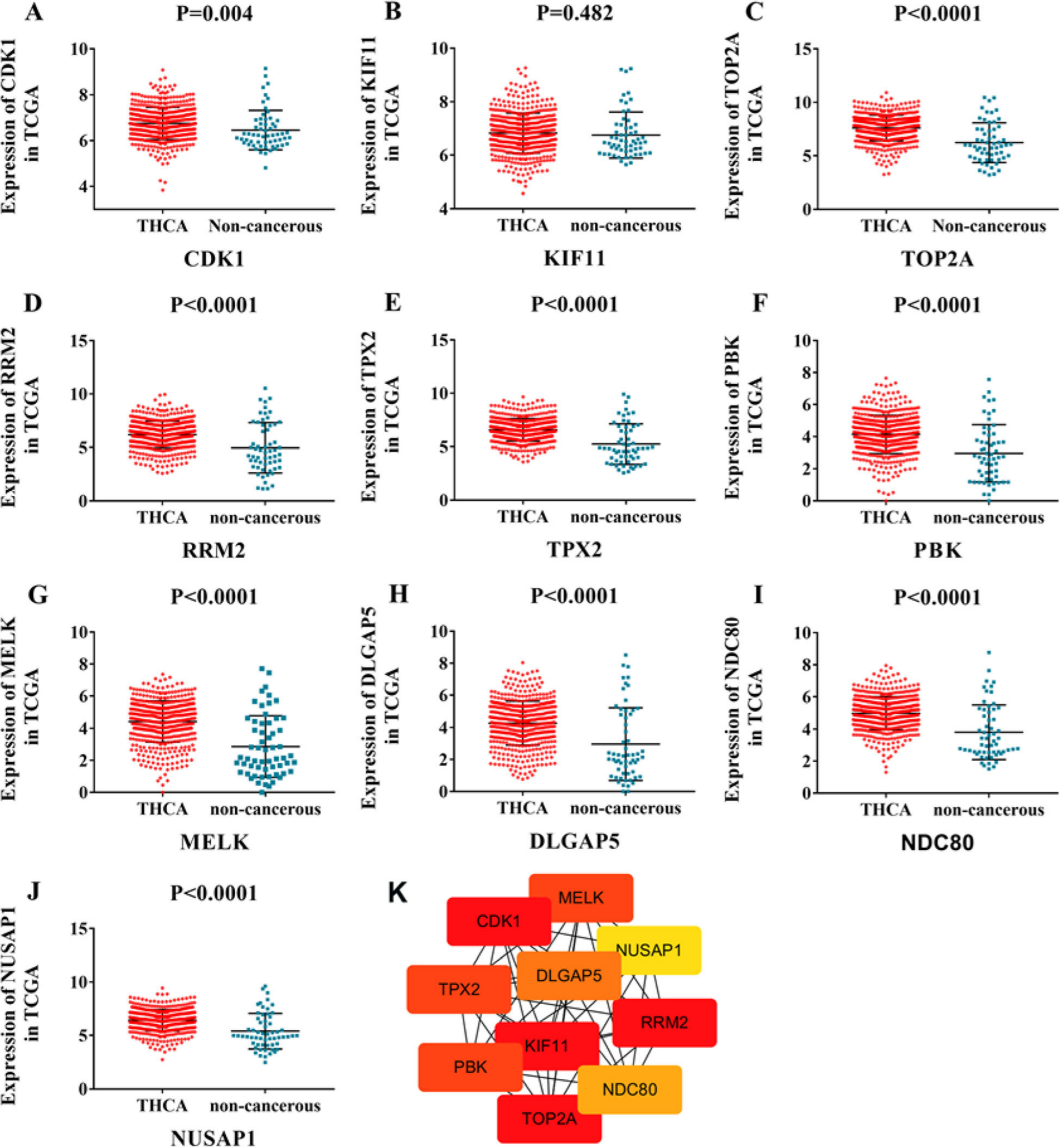
Hub genes of BSG in TC. **a**–**j** Scatter plots of top 10 hub genes in thyroid cancer (TC) based on RNA-seq data. **a** Cyclin dependent kinase 1 (CDK1). **b** Kinesin family member 11 (KIF11). **c** Topoisomerase (DNA) II alpha (TOP2A). **d** Ribonucleotide reductase regulatory subunit M2 (RRM2). **e** Microtubule nucleation factor (TPX2). **f** PDZ binding kinase (PBK). **g** Maternal embryonic leucine zipper kinase (MELK). **h** DLG-associated protein 5 (DLGAP5). **i** Kinetochore complex component (NDC80). **j** Nucleolar and spindle associated protein 1 (NUSAP1). **k** Top 10 hub genes calculated by String and CytoHubba of Cytoscape based on the intersect genes

Using PPI analysis and CytoHubba, we identified the top 10 genes as hub genes of BSG in TC (Fig. 8k): cyclin dependent kinase 1 (CDK1), kinesin family member 11 (KIF11), topoisomerase (DNA) II alpha (TOP2A), ribonucleotide reductase regulatory subunit M2 (RRM2), microtubule nucleation factor (TPX2), PDZ binding kinase (PBK), maternal embryonic leucine zipper kinase (MELK), DLG-associated protein 5 (DLGAP5), kinetochore complex component (NDC80), and nucleolar and spindle associated protein 1 (NUSAP1).

The expression levels of 9 hub genes in TC tissues were markedly higher than in non-cancerous tissues except KIF11: CDK1 (6.7486 ± 0.70712 vs. 6.4577 ± 0.86347, *p* < 0.0001), TOP2A (7.6083 ± 1.19671 vs. 6.23 ± 1.86183, *p* < 0.0001), RRM2 (6.1999 ± 1.25053 vs. 4.9689 ± 2.35379), TPX2 (6.5675 ± 1.05049 vs. 5.2402 ± 1.88557, *p* < 0.0001), PBK (4.1448 ± 1.18686 vs. 2.9661 ± 1.78316, *p* < 0.0001), MELK (4.422 ± 1.27704 vs. 2.8595 ± 1.92385, *p* < 0.0001), DLGAP5 (4.2636 ± 1.36501 vs. 2.9592 ± 2.27261, *p* < 0.0001), NDC80 (4.988 ± 1.02333 vs. 3.8061 ± 1.70888, *p* < 0.0001), and NUSAP1 (6.4232 ± 1.00652 vs. 5.4004 ± 1.67015, *p* < 0.0001) (Fig. 8a–j, Supplementary Table S2). Corresponding AUCs for these hub genes were also obtained, including CDK1 (AUC = 0.6571), TOP2A (AUC = 0.741), RRM2 (AUC = 0.6911), TPX2 (AUC = 0.7392), PBK (AUC = 0.7275), MELK (AUC = 0.753), DLGAP5 (AUC = 0.7101), NDC80 (AUC = 0.738), and NUSAP1 (AUC = 0.7234) (all *p* < 0.0001) (Supplementary Figure S3, Supplementary Table S2).

## Discussion

This study had two main aims. One was to identify the change of basigin (BSG) expression in thyroid cancer (TC) by collecting all possible sources of data, including data from IHC, RNA-seq, microarrays, and literature. The other was to create a preliminary study of the possible underlying mechanisms of BSG in TC using co-expressed genes and DEGs. We collected 1182 TC cases and 437 non-cancerous cases (*n* = 17) from all possible sources, and the combined SMD for BSG was 0.39 (*p* < 0.0001). The AUC, which was used to assess the capacity of BSG to distinguish TC from non-cancerous tissues, was 0.6986. Subsequently, we found that BSG levels were upregulated in TC. We also performed GO and KEGG analyses for BSG co-expressed genes and DEGs, and the results showed that genes tended to enrich in terms which were corrected with the extracellular matrix (ECM). Therefore, we surmised that BSG plays a part in TC by means of infecting the ECM.

As we all know, TC is the most common endocrine malignancy. Moreover, present researchers who have studied BSG expression levels have shown that BSG plays an important part in tumor cell invasion, metastasis, and angiogenesis [40–44]. Previous studies tended to focus on the relationship between BSG and differentiation levels of TC [16, 17]. Nevertheless, few studies have contributed to understanding the relationship between BSG expression levels and TC. In this study, we constructed an analysis using a large sample gathered from all possible sources, including co-expressed genes and DEGs of BSG, which have never been done before. We sincerely hope that the results of this study will assist with the development of TC therapy and provide a new direction for further study of BSG and TC.

Few studies have focused on the expression levels of the BSG protein and mRNA in TC, and previous studies were more likely to focus on how the differentiation level of TC was linked with the positive expression of BSG and MMP. In addition, the sample sizes of these studies were small, and the methods were limited to IHC. Aratake et al. [16, 45] indicated that BSG expression correlated significantly with the degree of dedifferentiation of TC based on immunocytochemical analysis. More interestingly, they also observed MMP-2-positive expression in tumor cells and/or the adjacent tissue in all anaplastic carcinoma (ATC) cases, and the cytological atypia of cells with BSG positive expression was greater than  that in cells with negative BSG expression. Tan et al. [20, 21] found that BSG and MMP-2 were expressed mainly in cancerous lesions by means of IHC staining of 156 differentiated carcinoma (DTC) cases. More importantly, they also observed that the positive expression of these two markers was more likely to be positively linked to extrathyroidal invasion, lymph node metastasis, depth of tumor invasion, and distant metastasis. This leads us to believe that the expression of BSG may be useful to predict the prognosis of DTC patients. Huang et al. [18, 46] evaluated the expression of BSG in 200 TC specimens and 20 nodular goiter specimens using IHC. They suggested that cytoplasmic BSG expression levels were significantly higher in TC tissues than in nodular goiter tissues and significantly higher across different pathological stages, and that they closely correlated with lymph node metastasis and depth of tumor invasion.

In the current study, we collected 171 cases of TC tissues and 87 cases of non-cancerous tissues from the First Affiliated Hospital of Guangxi Medical University and analyzed the BSG expression profiles. In doing so, we discovered that the protein expression levels of BSG were significantly higher in TC cases based on the IHC experiments. The AUC of BSG expression was 0.985, and the corresponding sensitivity and specificity were 0.912 and 0.966, respectively. We also found that expression of the BSG protein tended to be statistically linked to cancer subtypes (*p* < 0.0001) based on the analysis of clinical parameters. However, the number of undifferentiated thyroid carcinomas (UTC) was only 2. Therefore, the more specific relationship between BSG expression levels and differentiation levels of TC needs further study and validation. More importantly, we also analyzed BSG expression profiles using 1182 TC cases and 437 non-cancerous cases (*n* = 17) collected from all possible sources, including IHC, RNA-seq, and microarrays. The combined SMD for BSG expression was 0.39, the diagnostic odds ratio was 3.69, and the AUC of the sROC curve was 0.6986. Making use of 505 TC cases from RNA-seq based on the Kaplan-Meier survival curve analysis, we found that TC patients with high BSG expression levels tended to have slightly worse rates of disease-free survival. However, the number of cases with BSG mRNA expression level and disease-free time was inadequate and accounted for a small proportion. Therefore, the survival analysis had a large bias. And the prognostic ability of BSG to predict survival needs to be checked out by a large amount of cases and further validated.

Previous studies have shown that BSG played an important role in cancers, but which mechanisms it used to promote the occurrence and development of TC? A recent study [46] demonstrated that BSG was a cofactor of TNF receptor-associated factor 6 (TRAF6). Shi et al. [47] indicated that TRAF6 and BSG assisted with BSG-mediated MMP9 could form the TRAF6/CD147/MMP9 pathway and was associated with angiogenesis and metastasis in ATC. Omi et al. [19] suggested the involvement of BSG in the invasiveness of follicular carcinoma (FTC) cells via regulation of MMPs based on immunoblot and IHC techniques. In addition, they indicated that upregulated expression of BSG in FTC was driven by epidermal growth factor (EGF) via the PI3K, ERK, and JNK pathways. Furthermore, a previous study [48] also found that the upregulated expression of BSG directly contributed to tumor angiogenesis by stimulating vascular endothelial growth factor (VEGF) production via the PI3K/AKT pathway. Li et al. [17] revealed that silencing of BSG in the TT cells in medullary carcinoma (MTC) could drive the inhibited proliferation of TT cells and the alteration of the cell cycle. Huang et al. [49] first confirmed that BSG took part in glycolysis and the transmembrane transport of lactate assisting with monocarboxylate transporters 1 (MCT1), a facilitator of lactate transport. This resulted in the decrease of extracellular pH and tumor progression. Fanelli et al. [50] revealed that Thyroid Stimulating Hormone (TSH) further upregulated the MCT/BSG complex, which was necessary for BSG expression, and assisted BSG translocation to the cytoplasmic membrane. In addition, Huang et al. [18] indicated that miR-125a-5p functioned as a tumor suppressor in TC by directly targeting and repressing BSG expression, thereby inhibiting aerobic glycolysis and subsequently suppressing cancer cell viability, migration, and invasion.

In the present study, we constructed GO and KEGG clustering analyses of 1299 candidate BSG co-expressed genes and DEGs in TC. And these BSG co-expressed genes and DEGs tended to enrich in pathways which were associated with ECM, cell adhesion, cell-cell interactions, and angiogenesis. In the KEGG pathway analysis, genes were more likely enriched in the following terms: ECM-receptor interactions, complement and coagulation cascades, *Staphylococcus aureus* infections, the p53 signaling pathway, and in protein digestion and absorption. At the same time, the thyroid hormone synthesis pathways, pathways in cancers, and the PI3K-Akt signaling pathway were also evident. Due to these results, BSG, a member of the immunoglobulin superfamily, was determined to be a potential stimulator of MMP. It stimulated MMP-mediated ECM degradation, cell adhesion, and cell-cell interactions and played a crucial part in tumor cell invasion [9, 51–54]. BSG could also promote tumor angiogenesis via regulating MMPs and VEGF. Additionally, many previous researchers have studied the relationship between BSG and human cancers and further confirmed the role of BSG in the development of human tumors. For example, Li et al. [55] indicated that BSG promoted cell proliferation, metastasis, and invasion in breast cancer via accommodating the expression of MMP-9 and VEGF. Qiao et al. [43] revealed that BSG expression was significantly upregulated in malignant bone cancer. Moreover, the expression of TRACP, MMP-2, MMP-9, and c-Src (osteoclast specific genes) was analyzed by RT-PCR. In a word, BSG promoted the cancer cell invasion and development of TC via regulating MMPs and affecting ECM. This was also true of the PI3K-Akt signaling pathway.

The important role played by BSG in tumor cell invasion made this gene an excellent target for cancer treatment. At present, there are options for targeted therapy of TC. Based on the immune response to specific antigenic peptides present on the tumor surface or cytoplasm, several immunotherapeutic strategies have been developed. For instance, chimeric antigen receptor (CAR)-T cell therapy targeting intercellular adhesion molecule (ICAM)-1 was preclinically validated in TCs, especially in PTC and ATC [56]. Capdevila et al. demonstrated the effectiveness of blocking checkpoints with programmed death-1 (PD-1)/PD ligand 1 (PD-L1) axis inhibitors in ATC [57, 58]. Cytotoxic T-lymphocyte-associated protein 4 (CTLA-4) could also be utilized in TC therapy. Furthermore, chemokine receptor-targeted immunotherapy for thyroid cancer was also worthy of attention, since previous studies have found that chemokines and their receptors such as CXCR1, CXCR2, CXCR3, CXCR4, CXCR7, DARC, CCR3, CCR6, CCR7, and CXCL5-CXCR2 axis play an important role in determining the cellular immunophenotype of thyroid tumor microenvironment [59]. Moreover, some novel drugs were utilized to TC therapy. For example, apatinib could induce apoptosis and autophagy of PTC cells through PI3K/Akt/mTOR signaling pathway [60]. Previous studies have indicated that emodin inhibited the angiogenesis and metastasis of ATC by regulating TRAF6-mediated pathways, including the TRAF6/CD147/MMP9 pathway [47]. Some studies have provided evidence that downregulation of BSG via lentivirus vector-based RNAi decreased cell proliferation, matrigel invasion, and tumor formation in breast cancer, especially in MCF-7 breast cancer cells [61, 62]. Zhang et al. [63] provided evidence that chimeric antigen receptor T cells induced by doxycycline targeting BSG could be used in the treatment of liver cancer. Another study also found that doxycycline inhibited the proliferation of gallbladder cancer cells by downregulating the expression levels of BSG and induced an early apoptosis response in cancer cells [64]. In addition, Wang et al. [65] found that metuzumab could inhibit metastasis of esophageal cancer via blocking the function of BSG. Furthermore, BSG inhibitors are now being developed and tested [66–68], and we hope our study can provide some evidence for BSG targeted treatment of TC.

There were still some limitations in this study. First, only 11 TC cell lines were collected from the CCLE database. Second, the insufficiency of RT-qPCR analysis was also evident. Besides, the number of cases with BSG mRNA expression levels and disease-free time was limited making the survival analysis have a large bias. The current results of survival analysis need to be checked out. Most importantly, the specific mechanisms underlying BSG in TC need further validation in vitro and in vivo.

## Conclusion

In this study, we demonstrated the upregulation of BSG expression in TC using meta-analysis and statistical analysis on the data collected from all possible sources, including IHC experiments, RNA-seq data, and microarray data. More importantly, we showed that BSG levels were closely correlated with the progression of TC may by affecting the ECM, cell adhesion, and cell-cell interactions. We hope further study can be performed to discover the specific molecular machinery of BSG that promotes the biological aggressiveness of TC

## Supplementary information

**Additional file 1: Supplementary Figure S1.** Integrated assessment of the BSG mRNA level in thyroid cancer (THCA) based on gene microarray. A: Forest plot; B: Sensitivity; C: Funnel plot (Begg’s test); D: Funnel plot (Egger’s test). **Supplementary Figure S2.** Expression level of BSG confirmed using sROC curves using microarray data. A: Sensitivity; B: Specificity; C: Positive likelihood ratios; D: Negative likelihood ratios; E: Diagnostic odds ratio; F: AUC of sROC. **Supplementary Figure S3.** Roc curve of top 10 hub genes in thyroid cancer (THCA) based on RNA-seq data. A: Cyclin dependent kinase 1 (CDK1); B: kinesin family member 11 (KIF11); C: topoisomerase (DNA) II alpha (TOP2A); D: ribonucleotide reductase regulatory subunit M2 (RRM2); E: microtubule nucleation factor (TPX2); F: PDZ binding kinase (PBK); G: maternal embryonic leucine zipper kinase (MELK); H: DLG associated protein 5 (DLGAP5); I: kinetochore complex component (NDC80); J: and nucleolar and spindle associated protein 1 (NUSAP1). **Supplementary Table S1.** Clinical pathological parameters and BSG expression in THCA data from IHC. **Supplementary Table 2.** The scores and expression of Top 10 hub genes in THCA

## Data Availability

The datasets generated and/or analyzed during the current study are available in the TCGA repository, https://cancergenome.nih.gov/, and the GEO repository, https://www.ncbi.nlm.nih.gov/geo/. The IHC data that support the findings of this study are available from Fanpu Biotech, Inc. (Guilin, China) but restrictions apply to the availability of these data, which were used under license for the current study, and so are not publicly available. Data are however available from the authors upon reasonable request and with permission of Fanpu Biotech, Inc. (Guilin, China).

## Abbreviations

ATC: Anaplastic carcinoma
AUC: Area under the curve
CAR: Chimeric antigen receptor
CCLE: Cancer Cell Line Encyclopedia
CI: Confidential interval
CTLA-4: Cytotoxic T-lymphocyte-associated protein 4
DAVID: Database for Annotation, Visualization, and Integrated Discovery
DEGs: Differentially expressed genes
DOR: Diagnostic odds ratio
DTC: Differentiated carcinoma
ECM: Extracellular matrix
FTC: Follicular carcinoma
GEO: Gene Expression omnibus
GO: Gene Ontology
GO_BP: GO biological process
GO_CC: GO cellular component
GO_MF: GO molecular function
HCC: Hepatocellular carcinoma
IHC: Immunohistochemistry
KEGG: Kyoto Encyclopedia of Genes and Genomes
MMPs: Matrix metalloproteinases
MTC: Medullary carcinoma
NDC80: Kinetochore complex component
PD-1: Programmed death-1
PD-L1: PD ligand 1
PPI: Protein-protein interaction
PTC: Papillary carcinoma
RNA-seq: RNA-sequencing
RT-qPCR: Real-time quantitative polymerase chain reaction
SMD: Standard mean difference
SD: Standard deviation
SRA: Sequence Read Archive
sROC: Summary receiver operating characteristic
SP/NK-1R: Substance P/neurokinin-1 receptor
STRING: Search Tool for the Retrieval of Interacting Genes
TCGA: The Cancer Genome Atlas
TC: Thyroid cancer
TSH: Thyroid stimulating hormone
UTC: Undifferentiated thyroid carcinoma
VEGF: Vascular endothelial growth factor
