# Supplementary material for: Immunohistochemical basigin expression level in thyroid cancer tissues
Source: World J Surg Oncol. 2020 Sep 5;18:240. doi: 10.1186/s12957-020-01975-9 (PMC7487720; doi:10.1186/s12957-020-01975-9)
Supplement: Supplementary file 1 — Additional file 1: Supplementary Figure S1. Integrated assessment of the BSG mRNA level in thyroid cancer (THCA) based on gene microarray. A: Forest plot; B: Sensitivity; C: Funnel plot (Begg’s test); D: Funnel plot (Egger’s test). Supplementary Figure S2. Expression level of BSG confirmed using sROC curves using microarray data. A: Sensitivity; B: Specificity; C: Positive likelihood ratios; D: Negative likelihood ratios; E: Diagnostic odds ratio; F: AUC of sROC. Supplementary Figure S3. Roc curve of top 10 hub genes in thyroid cancer (THCA) based on RNA-seq data. A: Cyclin dependent kinase 1 (CDK1); B: kinesin family member 11 (KIF11); C: topoisomerase (DNA) II alpha (TOP2A); D: ribonucleotide reductase regulatory subunit M2 (RRM2); E: microtubule nucleation factor (TPX2); F: PDZ binding kinase (PBK); G: maternal embryonic leucine zipper kinase (MELK); H: DLG associated protein 5 (DLGAP5); I: kinetochore complex component (NDC80); J: and nucleolar and spindle associated protein 1 (NUSAP1). Supplementary Table S1. Clinical pathological parameters and BSG expression in THCA data from IHC. Supplementary Table 2. The scores and expression of Top 10 hub genes in THCA [file 12957_2020_1975_MOESM1_ESM.docx]

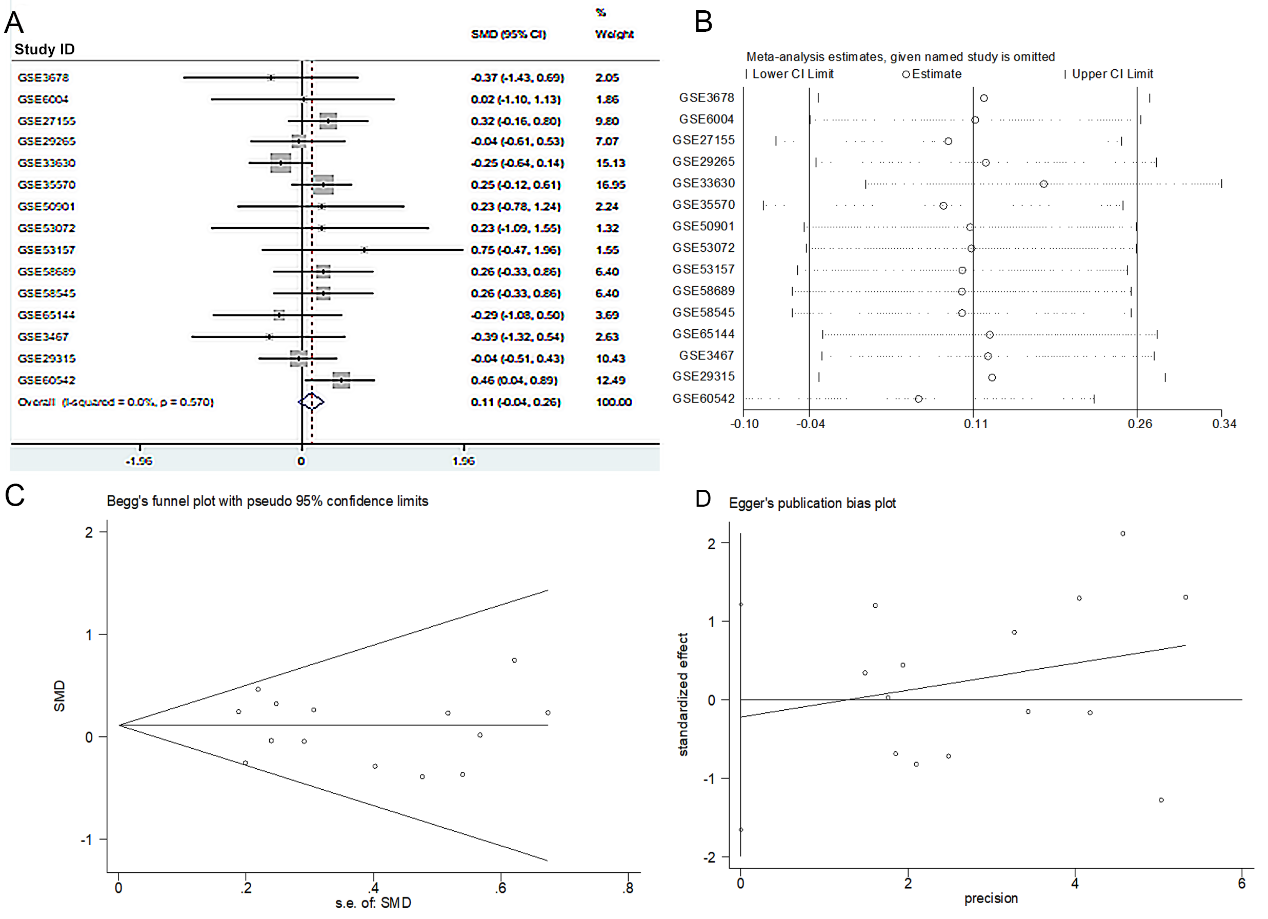


Supplementary Fig.1. Integrated assessment of the BSG mRNA level in thyroid cancer (THCA) based on gene microarray. A: Forest plot; B: Sensitivity; C: Funnel plot(Begg’s test); D: Funnel plot(Egger’s test)


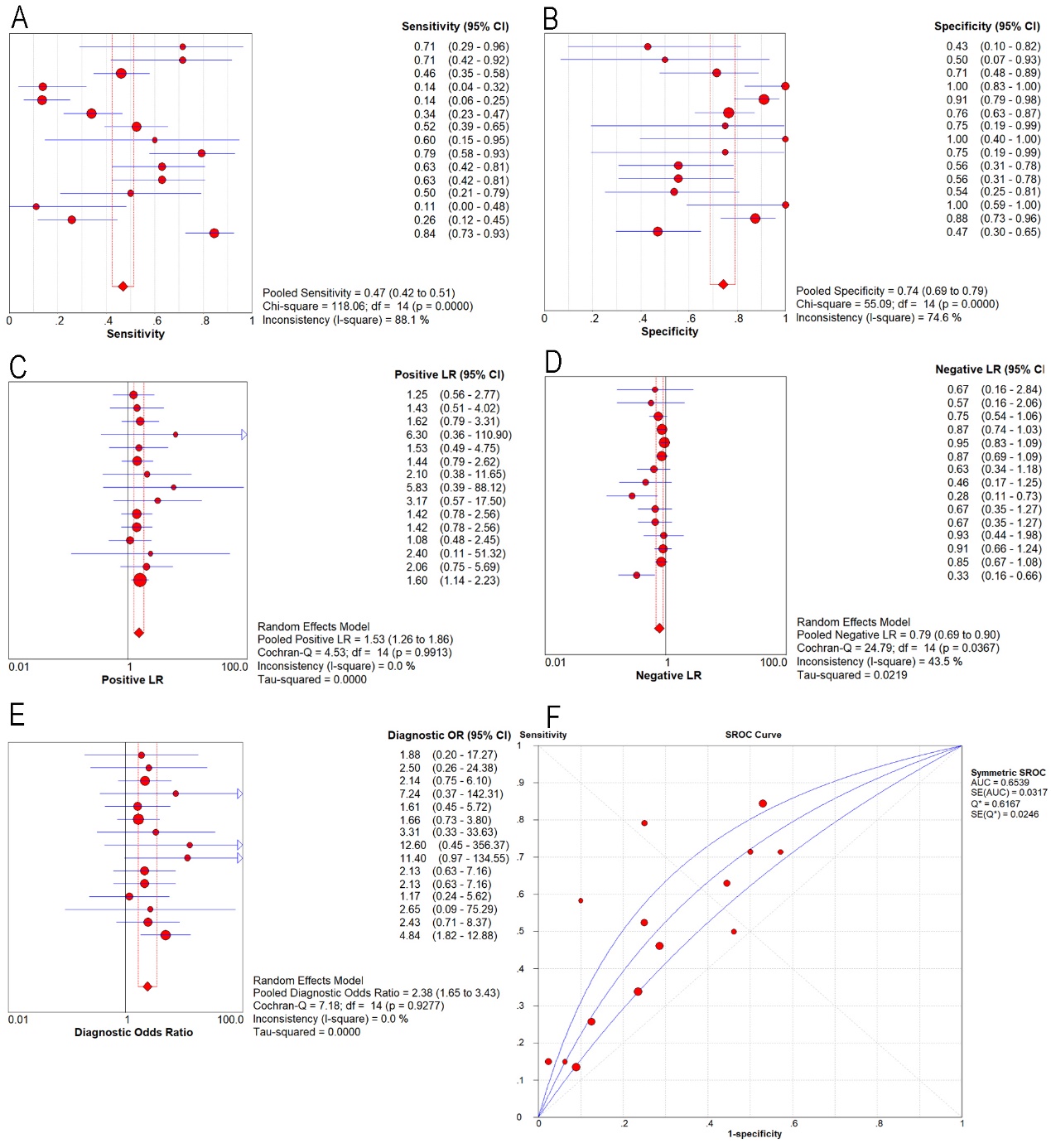


Supplementary Fig. 2. Expression level of BSG confirmed using sROC curves using microarray data. A: Sensitivity; B: Specificity; C: Positive likelihood ratios; D: Negative likelihood ratios; E: Diagnostic odds ratio; F: AUC of sROC.


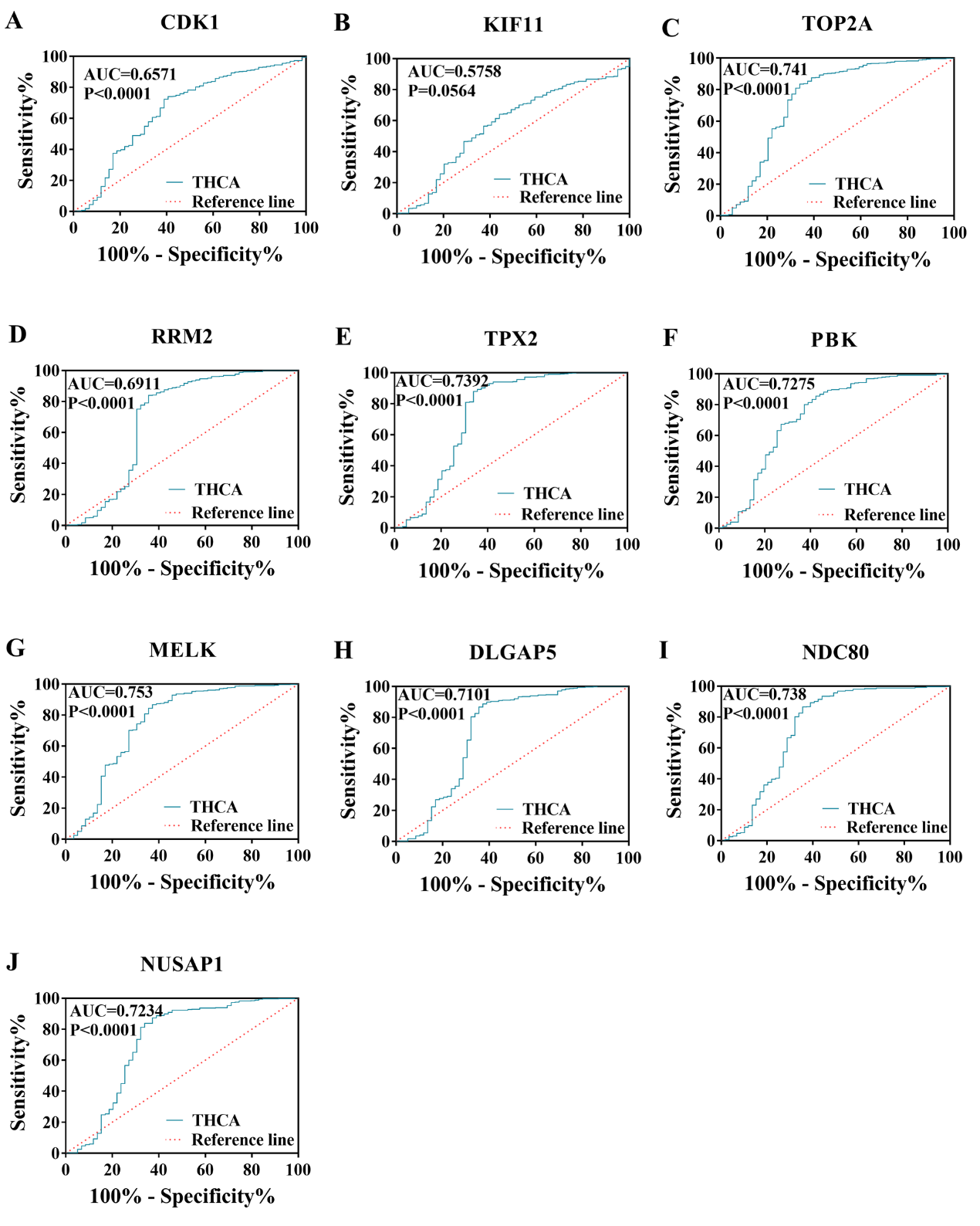


Supplementary Fig. 3. Roc curve of top 10 hub genes in thyroid cancer (THCA) based on RNA-seq data.

A: Cyclin dependent kinase 1 (CDK1); B: kinesin family member 11 (KIF11);

C: topoisomerase (DNA) II alpha (TOP2A); D: ribonucleotide reductase regulatory subunit M2 (RRM2); E: microtubule nucleation factor (TPX2); F: PDZ binding kinase (PBK); G: maternal embryonic leucine zipper kinase (MELK); H: DLG associated protein 5 (DLGAP5); I: kinetochore complex component (NDC80); J: and nucleolar and spindle associated protein 1 (NUSAP1).

Supplementary Table 1 Clinical pathological parameters and BSG expression in THCA data from IHC

| Characteristic |  | expression of BSG | | | |
| --- | --- | --- | --- | --- | --- |
|  |  | n | mean±SD | t/F value | p value |
| Tissue | THCA | 171 | 7.6±1.366 | 29.237^a^ | <0.0001 |
|  | non-cancerous | 87 | 1.32±1.749 |  |  |
|  |  |  |  |  |  |
| Gender | Female | 117 | 7.65±1.367 | -0.747 | 0.456 |
|  | Male | 54 | 7.48±1.37 |  |  |
|  |  |  |  |  |  |
| Age | >=60 | 41 | 7.3659±1.5930 | -1.111 | 0.271 |
|  | <60 | 130 | 7.6692±1.2843 |  |  |
|  |  |  |  |  |  |
| Pathologic T | T1 | 14 | 7.5±1.45444 | 1.022^b^ | 0.384 |
|  | T2 | 95 | 7.5158±1.45033 |  |  |
|  | T3 | 56 | 7.8214±1.14586 |  |  |
|  | T4 | 6 | 7.0±1.67332 |  |  |
|  |  |  |  |  |  |
| T | T1-T2 | 109 | 7.5138±1.44411 | -1.102 | 0.272 |
|  | T3-T4 | 62 | 7.7419±1.21379 |  |  |
|  |  |  |  |  |  |
| Pathologic N | N0 | 129 | 7.5349±1.41447 | -1.034 | 0.303 |
|  | N1 | 42 | 7.7857±1.20032 |  |  |
|  |  |  |  |  |  |
| Pathologic M | M0 | 170 | 7.5941±1.36956 | -0.295 | 0.768 |
|  | M1 | 1 | 8 |  |  |
|  |  |  |  |  |  |
| Subtype | Differentiated carcinoma^c^ | 169 | 7.5917±1.37327 | -0.419 | 0.676 |
|  | Undifferentiated carcinoma | 2 | 8±0 |  |  |

a. Student's 2-sample independent t-test was applied.

b. One-way analysis of variance (ANOVA) test was conducted.

c: Differentiated carcinoma including Papillary carcinoma (PTC), Follicular carcinoma (FTC) and Medullary carcinoma (MTC).

THCA: thyroid cancer.

Supplementary Table 2 The scores and expression of Top 10 hub genes in THCA

| Gene | Rank | Score | Gene expression | | | ROC curve | | |
| --- | --- | --- | --- | --- | --- | --- | --- | --- |
|  |  |  | p value | THCA | non-cancerous | P‑value | 95% CI | AUC |
| CDK1 | 1 | 3.24E+25 | 0.004 | 6.7486± 0.70712 | 6.4577± 0.86347 | <0.0001 | 0.5754 - 0.7389 | 0.6571 |
| KIF11 | 2 | 3.24E+25 | 0.482 | 6.8268± 0.75194 | 6.7528± 0.86438 | 0.0564 | 0.4959 - 0.6558 | 0.5758 |
| TOP2A | 3 | 3.24E+25 | <0.0001 | 7.6083± 1.19671 | 6.23± 1.86183 | <0.0001 | 0.6554 - 0.8266 | 0.741 |
| RRM2 | 4 | 3.24E+25 | <0.0001 | 6.1999± 1.25053 | 4.9689± 2.35379 | <0.0001 | 0.5931 - 0.7892 | 0.6911 |
| TPX2 | 5 | 3.24E+25 | <0.0001 | 6.5675± 1.05049 | 5.2402± 1.88557 | <0.0001 | 0.6472 - 0.8311 | 0.7392 |
| PBK | 6 | 3.24E+25 | <0.0001 | 4.1448± 1.18686 | 2.9661± 1.78316 | <0.0001 | 0.6424 - 0.8125 | 0.7275 |
| MELK | 7 | 3.24E+25 | <0.0001 | 4.422±1.27704 | 2.8595± 1.92385 | <0.0001 | 0.6696 - 0.8363 | 0.753 |
| DLGAP5 | 8 | 3.24E+25 | <0.0001 | 4.2636± 1.36501 | 2.9592± 2.27261 | <0.0001 | 0.616 - 0.8041 | 0.7101 |
| NDC80 | 9 | 3.24E+25 | <0.0001 | 4.988± 1.02333 | 3.8061± 1.70888 | <0.0001 | 0.6479 - 0.828 | 0.738 |
| NUSAP1 | 10 | 3.24E+25 | <0.0001 | 6.4232± 1.00652 | 5.4004±1.67015 | <0.0001 | 0.6331 - 0.8138 | 0.7234 |
